# Reparations not remuneration: Redefining the future of lived experience

**DOI:** 10.1371/journal.pmen.0000495

**Published:** 2025-11-20

**Authors:** Parth Sharma

**Affiliations:** 1 Program Associate and Consultant, Sangath, Bhopal, India; 2 Mentor and Consultant aves Mental Health (Global Mental Health Peer Network), Bhopal, India; PLOS: Public Library of Science, UNITED KINGDOM OF GREAT BRITAIN AND NORTHERN IRELAND

*Abolition* will not be found in an academic essay.

What you may find here instead is a critical reflection of the current ecosystem of the Mental Health Industrial Complex and radical ideas around Lived Experience that elicit one recurring emotion: discomfort.

## Introduction

### Lineage statement

A lineage statement is the recognition of whose voice informs this text and where the voice comes from. It is an invitation to the reader to henceforth engage in this dialogue.

I am Parth Sharma (he/they), a Queer, Non-Binary, Mad person. Each of those words brings with them histories of movements towards collective liberation. For the longest time the cis-hetero patriarchal system used Queer and Mad as stigmatizing labels against those that did not fit the normative presentation of gender and sanity. Today as a person with intersectional lived experience, I am conscious of their usage and ascribe them to my lineage as an act of reclamation and resistance [1].

While there are parts of my identity that cause me marginalization, I also recognize the immense privilege I operate with of being a young, upper caste, light-skinned, person from mainland India [2]. Today as I write this essay in English, I urge you to read, reflect and act on these words in the way you deem fit.

This Essay aims to disrupt the way we view lived experience, and I challenge readers to join me as accomplices in the cause. My words and work are routinely labeled as loud and often extreme. However, I maintain that the discomfort my writing causes in sanitized spaces of academia is incomparable to the harm our communities have experienced across decades. Discomfort is a byproduct of progress and change, and I invite you to reflect on that.

These experiences are not a monolithic representation of liberation, abolition, or lived experience, though I invite you to find spaces of resonance and reflection within them. If you find yourself uncomfortable reading through the urgency of this text, I ask that you sit with and recognise that discomfort and challenge the systems you participate in, if not profit from, on a daily basis.

As a person that operates on reasonable accommodations and routine accessibility needs, I am cognizant of the fact that academia as an institution perpetuates strategic exclusion. I write with the awareness that this text in many ways (length or language) will remain inaccessible. Keeping that in mind, I am actively working on creating open-access, visual, plain language and conversational spaces to better represent these ideas and honour our communities.

## *Reparations* is not a scary word

At its very core, the principle of reparations is atonement. Atonement for the historical wrongdoings of psychiatry and the acknowledgment by those who continue to profit from these wrongdoings today. Often conflated as a tool of empowerment and confused with an arbitrary sum of capital measured against an experience of systemic violence, reparations continue to be a conversation that the global movement for mental health avoids, if not suppresses as somewhat of a *scary word.*

The world of psychiatry has made considerable progress over the last few years to understand and embed the term *Lived Experience* within its nomenclature. Be it academia, practice or policy; today Lived Experience of Mental Health in many ways is understood to be an equally important stakeholder as the technical expertise of service providers and academics [3].

When I think of this progress, I am both thankful and enraged. The former is an ode to the decades of critical and anti-psychiatry, mad pride, disability justice and service user movements across the world that initiated this change [4,5]. The latter as I watch, the language and labor of these movements, be co-opted, extracted and discarded at the wills of people with institutional power. Yes, progress has been made to give *us* a seat at the decision-making table, although is there acknowledgement of the very violent colonial legacy that formed that table in the first place? How can conversations around repair occur when people with lived experience are still a tick mark on an equity checkbox?

Today as I watch my colleagues and comrades rally the grounds demanding pay parity and remuneration for Lived Experience work, I am reminded that in many ways we are not only asking for a formal recognition of our labor by the very people that have historically harmed us, but we are demanding that our humanity be recognized within a very unjust system. A system that deemed us not too long ago, as sub-humans and justified its superiority through eugenics, racial capitalism and a narrow colonial perception of science.

In a way, remuneration for labor does help inclusion of Lived Experience voices, representation in panels, boards and consultations, and furthers the cause that was painstakingly built over decades. Although in many ways, it also reduces lived experience to a commodity that can be bought and sold, further rewarding those that can *sell their oppression* in a palatable way to achieve this formal recognition for their labor.

What is scarcely ever spoken of is the privilege to remain activated. Activated enough to participate in advocacy, narrate experiences and provide professional expertise. Let alone the geographical, caste, class, racial and gendered impact of being present enough to be marketable and thus *demand* value for labor. This goes beyond representation politics of visibility to actively question erasure in the Lived Experience movement of an entire people. What of those that are actively incarcerated, chronically ill, disabled or unhoused? What of those that do not speak the required language to participate in panels, boards and consultations? What of those who simply do not fit the box neatly designed for our current commercial understanding of Lived Experience?

The answers to these questions lie in understanding the need for reparations and the shift from our current model of Lived Experience Inclusion.

The words of author Ijeoma Oluo come to mind, who in her book ‘*Mediocre: The Dangerous Legacy of White Male America’*, uses the phrase ‘*works according to design’* to describe oppression in the real world [6]. It reaffirms that oppression was not an outcome of a faulty system, but rather the very framework used to design social institutions as we know it today.

The very institution and industry of mental health is oppressive by design. Partly due to its reductive history of biological determinism [4]. And partly because those that designed these structures and now chair their reform are people who have benefited from these injustices and continue to profit from them. This phrase ‘*works according to design’* also encapsulates the very contradiction I described around the remuneration model of Lived Experience, where the oppressive system demands commodification of lived experience through its core design. Thus, ensuring that reform works within the feasibility limits of the oppressive system.

It is here that I implore you to sit with the discomfort you feel, if at all, as you approach the idea of reparations. I urge that you go beyond logical fallacies and thought-ending cliches that are used in sanitized spaces to evade critical thinking and approach this conversation with intentionality. I ask that you think of the *why*, before the *how*, however keeping both with you as you continue engaging with this Essay.

*Reparations* is not a scary word. It is a process of acknowledgement that demands accountability for decades of historical trauma, and the relentless profiteering done by academics, researchers and practitioners at the expense of those that were and continue to be harmed by psychiatry. Moreover, the conversation around reparations extends to transforming laws and systems, where reparations are a medium towards necessary structural change such as deinstitutionalization and prohibition of coercive practices. Once we understand *why* reparations are justified and needed, the discussions around *how* feasible they are in a growingly polarizing world become tangible. The biggest obstacle I imagine is not describing the process of reparations but asking *you* the reader to imagine the necessity for reparations in the first place.

## Mental Health Industrial Complex (MHIC)

### Mental Health Industrial Complex

Refers to a network of oppressive institutions, corporations, government or non-government agencies, and practicing bodies that profit from, regulate, and perpetuate the bio-medical mental health system.

Our perceptions of Mental Health have been borrowed from ableist, eurocentric, cisgendered, and heterosexual principles [7]. Systems that have historically and continue to presently be deep rooted in white supremacy guide the ways through which Black, Brown and Indigenous bodies engage with research, practice and policy. These perceptions form the very foundations of the Mental Health Industrial Complex. The Mental Health Industrial Complex (MHIC) is a severely under-researched yet pervasive structure that comprises health systems that provide care, the state’s necropolitical paradigm on ‘the people fit to save’ and the private entities that capitalize over systemic oppression [8]. It does not operate in isolation and is closely intertwined with the carceral state, rampant militarization, the prison industrial complex and other systemic forms of social control, [9]. These structures compound harm for individuals, especially those from marginalized groups, by reinforcing the very social determinants of health that exacerbate distress such as poverty, discrimination, and state violence. It is imperative that we note that the Mental Health Industrial Complex is politically motivated to ensure that the oppressed can only look inward while the dominant social class contributes to their oppression and operates through blissful ignorance of their role. This specific ontology of subjectivity, created through the invention of institutional colonial psychiatry, goes on to define the entire human experience of survival as a result of brain chemistry, which stands as not only a restrictive lens but also a blatant colonial project [10].

Acts of liberation have been pathologized by western colonial psychiatry for as long as one can remember. Insurgent Blackness, Rebellion by enslaved people, Feminist principles of autonomy, Anticolonial Resistance and Queer and trans rights are a few of the many deviations that have threatened the Empire and thus been pathologized [11–14]. All forms of policing engage in a necropolitical control over the bodies of the most disenfranchised. Policing carries with it an unnecessary violence and coercive control that threatens the masses into acting in compliance with the state. The state decides which bodies get arrested, displaced, gentrified, starved and overreported, and those that police carry out the use of force and overhanging fear to move communities away from communal sovereignty and towards racial capitalism. The necro-economy that drives this policing works at the expense of the oppressed [15,16].

The British colonial powers in South Asia, particularly India, are evidence to how this necropolitical ideology was used to control and oppress. History is a testament to the stories of enslavement, and human experimentation in colonial asylums, where British colonists routinely engaged in enlightened despotism to “cure the savage lunatic Indian”. After the 1857 revolution, anti-colonial sentiment was pathologized and resulted in the psychiatric incarceration of Indians as a matter of documented policy through the 1858 Indian Lunatic Asylums Act. Forced incarceration, medical coercion, restraint, forced treatment, and outright enslavement of those in asylums into rope making, masonry, and gardening, sustained the “asylum industries” as necessary interventions for the “Indian lunatics.” Not only did the British colonial administration create a necro-economy, but they also ensured the enslavement of Indians in psychiatric facilities sustained it for the profit of the East India Company and the British Raj [17]. Survivor narratives from existing, albeit renamed asylums in India today may very well attest to this phenomenon continuing even today.

The Mental Health Industrial Complex runs just like a prison. It is built on exploitation and has been co-opted by private entities that are intertwined with the carceral state, often resulting in the increased psychiatric incarceration of people with lived experience that resist the status quo.

## Commodification of lived experience

Let’s consider the following thought example to better understand our own positions within the Mental Health Industrial Complex.

The Empire that formed the Mental Health Industrial Complex is like a factory, rooted in the fantasy of violence. Colossal at best; it operates like a Mirrorball, made up of many different broken pieces, angled perfectly to sell an illusion to those watching it spin in awe. Like any factory, it includes a few minds that build the product, and the rest of many overworked mouths that relay it along to you, the bystander. While most of the public is busy fending for a warm meal and some cold water, buying this product, shiny and new, seems impossible, yet the promise of having it ensures that they continue working hard. For the rest, many who buy, are rewarded and those who don’t are strong-armed into submission until the factory comes up with something new to sell. What do you do when the product you’re given is visibly fragmented, repackaged, and incomplete by design? More importantly, what do you do when you’re bound to manufacture this very product repeatedly?

When evaluating responsibility in consumer culture, where do we place accountability? Is it with the retailer that ensures the transaction, or the advertiser that influences the purchase and frames the fragmented, repackaged and incomplete product as desirable? In their work around Manufacturing Consent, Chomsky and Herman deconstruct the Empire through a propaganda model allowing us to understand the systemic, structural and interpersonal factors that govern communications, coercion and public narrative [18]. What stands out to me is this particular section where Chomsky writes for the need to tame the bewildered herd through the manufacture of consent. They continue by highlighting how in countries where power is situated in the hands of the state, we see a monopolistic media where the control is reinforced through state censorship to serve the interests of the elite [18].

Lived Experience in its current form resembles this *incomplete repackaged product* that is routinely sanitized to fit a perception of recovery and is digestible to both policy makers and funders. People with lived experience who remain “activated” enough to articulate their perspectives are repeatedly compelled to manufacture narratives that conform to pre-established frameworks of social acceptance. Not only does this expectation place a disproportionate burden on the oppressed to negotiate power; it also requires many to self-censor and comply with dominant norms to gain visibility. Rather than translating into meaningful change, lived experience often functions as a proxy for social impact. It routinely offers the appearance of progress without influencing the underlying factors that perpetuate oppression. Oppression then becomes a commodity in an industry that profits from depoliticizing reality and conditioning people to erase systemic, structural and interpersonal harm to be recognized, heard and if budgeted for then remunerated.

People with lived experience are by no means a homogenous group, while many peer-led organizations align themselves with dominant perspectives, there exists a heterogeneity represented by abolitionists, survivors and mad thinkers who are routinely neutralized and policed for their disruption of the status quo. This Essay critiques the institutional framing of lived experience and the “Empire” that sustains the industry. Importantly, the decision to remain activated and resist assimilation must be understood as nonlinear expressions of lived experience. This is to ensure that those that are courageous enough to speak out are not erased under the critiques of the factory for manufacturing their narratives or for their choice to retain visibility and proximity to sanitized peer-led spaces as a source of empowerment.

## Epistemic neocolonialism

It is always an inquiry worth making–whose voice is reflected in Mental Health the most, and why is some knowledge considered more important than others? These two questions are interrelated as the answer to both lies in the very notion of Epistemic Neocolonialism.

Epistemic Neocolonialism refers to the phenomenon where people from oppressed groups are systematically denied opportunities to create knowledge and derive meaning from their experiences. Rarely do the oppressed get opportunities to become “epistemic agents”, where they are creators of knowledge, that in turn excludes anyone from the narrative that does not have close proximity to the dominant status quo [19]. These ideas were first applied to instances of discrimination against women and people of color but have since come to refer to a wider set of issues under social justice [20].

Earlier in this Essay, I was referring to the pathologization of resistance with examples of Insurgent Blackness and Rebellion by enslaved people. To that account it is worth considering the example of Cartwright who practiced racial medicine and urged that psychiatry pathologize the “runaway slave escaping from their masters” as a clinical mental health illness called “drapetomania” [11,21]. Popular at that time, one may think this is no longer the case, and that counter narratives are surely present in literature. However medical racism and *modern day drapetomania* is alive and well even today as evidence to this very phenomenon [22].

The MHIC profits from social stratification and epistemic injustice and requires the oppressed to remain at their identity-determined location at all times– be it gender, caste, class, race or religion. This reaffirms the necropolitical paradigm and its capacity to ‘let people die,’ upheld by a revisionist logic that frames an oppressive *something* as better than a non-oppressive *nothing*.

### A good cop remains a cop

A fitting allegory for well-meaning practitioners in the Mental Health Industrial Complex, who aim to do good through their positions however ignore the power and agency they hold in making the choices to “do good”. Owing to the many from our community that are not here today and the few activated to speak about their lived experience and struggle to be recognized or remunerated, I urge you (with institutional power) to pause and examine your role in perpetuating the Mental Health Industrial Complex.

Is your time spent *working according to design*?

## We do not need more allies, we need abolitionists

I write this section feeling enraged and as a provocation for *you* the reader, as you may sit within the comfort of your allyship.

In many ways I consider the usage of ‘ally’ and in extension ‘allyship’ to be oppressive slurs used by the elite masking their co-optation of the marginalized. These allies include academics, scholars and practitioners who have and continue to capitalize on the commodification of liberatory praxis. Their *allyship* is dependent on gaining proximity to certain parts of our marginalized identities that can be exploited while never losing the relation they have with oppressive centers.

Allyship is an extension of the colonial power hierarchy. It is steeped in the fantasy of control over marginalized bodies. It borrows from the white savior narrative and assumes authority over ‘those in need of help’. Allies function with a romanticization of resilience which is almost obscene; they view the oppressed as helpless victims that must be rehabilitated. They are habituated to the disenfranchisement of the oppressed and actively work towards the ‘Other’ and drive pleasure out of their own efforts in activism and advocacy [23].

Allies, even the well-intentioned, may lack the hermeneutical resources required to engage in reflexive and power-aware work. While many simply act out of woeful ignorance, unaware of how deeply detached they are from the living reality of the oppressed, even as they cosplay as members of those communities. This proximity to liberation movements and the need to appropriate the aesthetics of violent resistance is almost a kind of performance. One that the ally carefully constructs, adopting a convenient persona to project an image of solidarity to the world.

In today’s increasingly risk averse society, maintaining this performance of allyship is necessary to climb the ladder of a career in social justice. A career built on the exploitation of marginalized bodies, and for the most part at their very exclusion. Many people have bestowed upon themselves with such benevolence, the title of *being an ally* to serve nothing but the illusion of *looking* good rather than *doing* any good.

Allyship is enough to win grants for community based ‘grassroots’ research, awards on inclusion and social currency for diversity. It is enough to intellectualize theory, create ‘discourse’ and participate in dissent. It is enough to sustain the attention economy of the news cycle and appease the oppressive elite that disproportionately controls the world’s capital.

Allyship is enough to manufacture consent.

### Performing Allyship: To be an ally is to be a wolf in sheep’s clothing

Allies portray a state sanctioned solidarity towards a cause they deem fit. They watch, examine, learn, scheme and understand which intersections within the community are ‘worth’ supporting. Here *worth* is defined not by the very existence and humanity of a person but by a narrow capitalist understanding of exploitable labor. Once identified, they strategically place themselves in proximity to these groups and co-opt their movement.

Today there exists an allyship for certain identities with Lived Experience. With *Nothing About Us, Without Us* and other popular sloganeering done in good faith this very model of allyship very conveniently leaves out those that are incarcerated, unhoused, serve as political prisoners, work as sex workers, are queer trans and elderly given the lack of funding/ publishing opportunities at these intersections. I implore you to locate this in the current news cycle of your region and answer these questions: who is the ally for the oppressed, where are they now and what is the relationship, they share with the state? This provocation is intended to help you lower the pedestal on which you reside as an ‘ally’ and intervene as, yet another movement is co-opted by the elite in service to the Empire.

I particularly caution against career activists that perform allyship.

These nonprofit capitalists continue to advance their professional portfolios as they build positions of power to perpetuate hierarchies within the communities they wish to spotlight and campaigns they wish to champion. Consider the example of a mental health non-profit organization in your region that has played a formative role in shaping rights-based advocacy. Within these organizations, one may encounter individuals who, despite being removed from the direct experience of systemic, structural and interpersonal violence, hold institutional power and technical expertise to conduct research and influence policy. These individuals may present themselves as well-intentioned allies committed to understanding the complexities of power, privilege, and positionality within their work. To legitimize their allyship, some may even pursue formalized education through courses, certifications and workshops offered by elite institutions to attain a symbolic social capital: a badge of allyship. This increases their credibility to continue their work without dismantling the power they hold and routinely benefit from. In doing so, they perpetuate a dynamic where their allyship supersedes the lived experience of the oppressed they research upon.

It is worth mentioning here that this critique of normalized power hierarchies within the Non-Profit Industrial Complex (NPIC) is not to further create an *us vs them* binary, however it is to spotlight the very skewed power dynamic that continues to exist and is routinely justified.

*“The particular threat to the intellectual today, whether in the West or the non-Western world, is not the academy, nor the suburbs, nor the appalling commercialism of journalism and publishing houses, but rather an attitude that I will call professionalism. By professionalism I mean thinking of your work as an intellectual as something you do for a living, between the hours of nine and five with one eye on the clock, and another cocked at what is considered to be proper, professional behavior-not rocking the boat, not straying outside the accepted paradigms or limits, making yourself marketable and above all presentable, hence uncontroversial and unpolitical and “objective.”*

― Edward Said, Representations of the Intellectual [24]

In *Representations of the Intellectual,* Edward Said spoke about the attitude of professionalism; the sanitised, marketable, objective and apolitical behavior where the ‘work’ of an intellectual happens within the constraints of the norm.

I extend this to allyship in Lived Experience work.

A badge of allyship then represents currency to purchase and sustain capital in the Non-Profit Industrial Complex where allies dictate the level of engagement within a struggle and ensure that *allyship* is inevitably tied to building a profit driven career. When this occurs, the *allies* who may or may not have joined the social justice sector to do good, are now dependent on harmful practices and performative inclusion to sustain their livelihood and that of their family. Livelihood at the expense of the oppressed. It is almost ironic to expect allies to recognize the harm they cause as many who do not operate on leadership positions struggle to make ends meet in a ‘cost of living crisis’ and watch as their allyship can only exist between the 9–5 from Monday to Friday. Capitalism fatigue by no means absolves the ally of this behavior; however, it does shed light on the very precarious nature of this industrial complex that devalues both the oppressed and their allies akin to the larger discursive phenomenon of ‘benevolent othering’ [25].

A note on how all oppressive systems interconnect with the earlier thought exercise of the factory. This commodification and objectification of care and support is a by-product of late-stage capitalism which functions on the ability to ensure that communities remain divided, and the onus of care remains carefully placed on the individual [26]. Those closer to the oppressive center then have a unique ability to co-opt the struggles of the marginalized through their savior narratives. In their career, allies work towards pulling the strategically underserved communities to the center through inclusion, training, diversity and equity requirements, and remuneration then becomes an easy gateway for this process and remains a socially sanctioned form of reform.

While many allies and their affiliated organizations now commend themselves for publishing ostensibly scientific literature on co-design, the discursive architecture of these efforts routinely betrays the very commitment they claim to uphold and enforce. Tokenistic inclusion of minority identities, extractive research, a lack of fair compensation and outright discrimination are a few of many ways through which those with institutional power continue to replicate the colonial and oppressive fabric of academic research, now through less oppressive methodologies.

By essentially employing certain members of a community within oppressive systems and having them operate as monolithic representations of an entire group, allies commodify a struggle and perpetuate the very oppressive hierarchies they stand against. This participatory assimilation conveys an epistemic injustice which is not limited to the lack of meaningful engagement and tokenization, but a more implicit coercion that conditionally shapes lived experience under existing frameworks and project assumptions [27]. Even within community-based research and ‘grassroots’ initiatives, radical projects that challenge the very framework of this colonial and capitalist system either find themselves sitting as discarded applications without funding or in need for revisions to satisfy the potential capitalist funders.

What is alarming is that allies in these systems do not face any tangible consequences from their actions and get to *log off* from their work at the end of the week, as people from the margins continue to live with the very real and tangible impact of systemic violence. As people with lived experience, our bodies are never at rest due to the very nature of these systems. Our oppression does not end between 9–5 from Monday to Friday or at the end of a grant cycle.

Co-optation of this nature also functions as a form of neo-liberalism where allyship thrives on a neutralizing dynamic by co-opting the original liberatory intent into a bland reformist agenda. In extension of the existing allies, people who have risen to positions of power from marginalized communities to now become Researchers, Mental Health Professionals and Career Activists also pick and choose language from resistance movements and repurpose the same as ways to undo the historical harm of psychiatry.

*“Dominant groups may adopt the language of marginalised groups and alter definitions of words over time, until terms like ‘empowerment’ and ‘peer’ become empty buzzwords or mean the opposite of what they once meant. The dominant group may selectively embrace parts of the less powerful group’s agenda and then water down these ideas so they become non-threatening and ineffective.”*

- Darby Penney and Laura Prescott in: Searching for a Rose Garden: Challenging Psychiatry, Fostering Mad Studies [28]

It is necessary to look at intersecting identities when examining this phenomenon. Predominantly able bodied, white, cisgendered, heterosexual, male dominated human rights organizations from the Global North countries ‘champion’ the cause of disability and mental health by operating at a proximity to the same oppressive center with one overarching goal: inclusion [7].By systematically isolating mental health to be a user-provider dynamic, not only do these organizations promote capitalism and the reduction of people into users/consumers; they ensure that their solutions to oppression are sanitised reforms rather than abolition.

## Beyond Allyship: Accomplices and Abolitionists

“*For the master’s tools will never dismantle the master’s house. They may allow us to temporarily beat him at his own game, but they will never enable us to bring about genuine change…I urge each one of us here to reach down into that deep place of knowledge inside herself and touch that terror and loathing of any difference that lives here. See whose face it wears. Then the personal as the political can begin to illuminate all our choices.”*

- Audre Lorde [29]

Where do we go past the idea of allyship? Indigenous Action offers a powerful framework for the abolition of what they describe as the “ally industrial complex”. They argue for the use of the term “accomplice” to deliberately provoke risk where the accomplice understands their role in collective liberation and acts with mutual consent and trust of the community. Unlike allies that rely on symbolic performances, accomplices are positioned to unsettle colonial systems through direct action. To be an accomplice is to commit a crime [30].

I ask *you*, the reader, to check in with yourself, locate your discomfort and then reflect on this excerpt from Lorraine Hansberry

*“Do I remain a revolutionary? Intellectually-without a doubt. But am I prepared to give my body to the struggle or even my comforts?… Comfort has come to be its own corruption”* [31].

Are you ready to step away from your allyship and join me as an accomplice in dreaming abolitionist futures? Are you ready to move past the idea of remunerations and discuss reparations?

## The future of Lived Experience: Beyond remuneration

As a mad disruptor with lived experience who was trained in providing clinical mental health support and has since divested from Psychiatry, it is crucial that I do not reduce this piece to a regurgitated opinion on the Mental Health Industrial Complex. At the same time, I wonder how we move past this capitalist idea of labor deep rooted in meritocracy and reimagine abolitionist futures. If the answer lies in decolonization, the following question comes to mind: Are we decolonizing a colonial system through diversity and social justice or are we looking to dismantle colonial systems of oppression entirely? [1,7].

Our current understanding of reparations comes from the ongoing work around reparations in the context of race, indigeneity, gender, caste, climate and conflict related movements [32–35]. Reparations at their core acknowledge and redress harm, and the call for reparations within psychiatry does not differ in value from these movements. In fact it builds on them and recognizes the interconnectedness between all movements for freedom and liberation.

Everyone with experience of human rights violation has a right to reparations [36]. Additionally, state parties that fail to prevent human rights violations have a legal obligation to provide

Reparations [37]. 164 states that are signatories of the *UN Convention On The Rights Of Persons With Disabilities (CRPD)* are currently integrating rights based mental health legislation within their national and regional context, focused on deinstitutionalization and community based mental health care [38]. Articles 13 and 19 of CRPD have each been interpreted to include reparations. In many ways the process of civil and political rights being reinstituted, and policies on affirmative action being implemented are signs of reparations being discussed

Within the context of lived experience, I speak of reparations to extend beyond just remuneration that is provided as income for labor. I speak of reparations that center the principles of disability justice: intersectionality, anti-capitalist politic, cross movement solidarity, interdependence and collective liberation [39]. Recognizing the tangible and redistributive measures that confront the enduring material consequences of colonial and psychiatric exploitation. Reparations that cover housing, medical support, healthcare, education, and utilities but also the creation of community-led infrastructures of care and healing that prioritize those historically pathologized and dispossessed due to systemic negligence and policy erasure. Reparations that take a two-pronged approach addressing the past harms and the systemic measures required to prevent further violence. As well as a moral, political, and ethical debt that is yet to be paid. The very acknowledgement of harm in formal and transparent ways by those who hold institutional power and profit from our experiences as they discard their allyship to become co-conspirators.

*Reparations are owed*, not because harm was done in an arbitrary past, quantified by colonial systems of measurement that commodify the worth of a human being, but because justice is at the core of repair. Justice that exists beyond the necropolitical paradigm of the Mental Health Industrial Complex and includes the incarcerated, queer, trans, unhoused and disabled among other groups that are strategically excluded and deemed disposable.

I reaffirm that the thesis of this essay is not concerned with *how* reparations are quantified, but rather with *why* reparations are necessary. While many of these ideas may be read as utopian, they already exist as tangible and ongoing realities in the form of affirmative action, reservations, and even emerging frameworks of psychiatric reparations [40,41].

*“The system will collapse if we refuse to buy what they are selling…their ideas, their version of history, their wars…their notion of inevitability. Remember this: We may be many and they may be few. Another world is not only possible, she is on her way. On a quiet day, I can hear her breathing”*

- Arundhati Roy [42]

The Mental Health Industrial Complex works to ensure that people with lived experience limit our perceptions of change to the narrow boundaries and existing feasibility frameworks of psychiatry. This Essay was in resistance to the MHIC and served as a deliberate provocation to re-imagine abolitionist futures outside the existing norms. While acknowledging that reparations are not a singular solution to the oppressive history of psychiatry, this provocation was aimed at eliciting discomfort in you, the reader. Discomfort that paves way for critical reflection to not only explore the *why* but to collectively dream about the *how* of justice and repair.

*Abolition* will not be found in this academic essay, although I hope intentionally engaging with this has allowed you some perspective on where to next look.
